# Stoma Acceptance Mediates Body Image Distress and Mental Health-Related Quality of Life: A Single-Center Study on Radical Cystectomy Patients with Ureterostomy

**DOI:** 10.3390/jcm13247682

**Published:** 2024-12-17

**Authors:** Benedetta Muzii, Francesco Di Bello, Claudia Collà Ruvolo, Simone Morra, Federico Polverino, Colomba Pessolano, Massimiliano Creta, Gianluigi Califano, Gabriele Pezone, Francesco Mangiapia, Pierluigi Alvino, Nicola Longo, Nelson Mauro Maldonato

**Affiliations:** 1 Department of Neurosciences, Reproductive Sciences and Odontostomatology, University of Naples “Federico II”, Claudia Collà Ruvolo, Via Sergio Pansini 5, 80131 Naples, Italy; benedetta.muzii@unina.it (B.M.); francesco.dibello@unina.it (F.D.B.); simone.morra@unina.it (S.M.); federico.polverino@unina.it (F.P.); massimiliano.creta@unina.it (M.C.); gianluigi.califano@unina.it (G.C.); gabriele.pezone@unina.it (G.P.); francesco.mangiapia@unina.it (F.M.); pierluigi.alvino@unina.it (P.A.); nicola.longo@unina.it (N.L.); nelsonmauro.maldonato@unina.it (N.M.M.); 2 Intradepartmental Program of Clinical Psychopathology, Federico II University Hospital, 80131 Naples, Italy

**Keywords:** body image, stoma acceptance, ureterocutaneostomy, mediation analysis, mental health, quality of life

## Abstract

**Background:** Muscle-invasive bladder cancer and subsequent radical cystectomy with ureterocutaneostomy significantly impact patients’ body image and quality of life, potentially increasing the risk of adverse mental health outcomes. Acceptance may represent a psychosocial resource to buffer the effects of body image impairment on health, thereby supporting stoma adjustment and preserving quality of life. **Objective:** This study aimed to investigate the mediating role of stoma acceptance in the relationship between body image distress and mental health. **Methods:** A single-center cross-sectional survey was conducted with 73 muscle-invasive bladder cancer patients undergoing radical cystectomy with ureterocutaneostomy. Participants completed structured, anonymous self-report measures assessing body image distress, stoma acceptance, and mental health-related quality of life through validated questionnaires. **Results:** Statistical analyses revealed significant negative correlations between body image distress and mental health and stoma acceptance. Conversely, stoma acceptance was significantly and positively associated with mental health. Regression-based mediation modeling indicated that stoma acceptance exerted a significant mediating effect on the relationship between body image and mental health-related quality of life. **Conclusions:** These findings highlight the considerable and unprecedented role of stoma acceptance as a mediating factor that may promote the adjustment and enhance the quality of life of urostomy patients. Further research is warranted to explore interventions targeting stoma acceptance to prevent body image distress and promote mental health.

## 1. Introduction

Bladder cancer (BC) is one of the most common neoplastic diseases worldwide, with 573,278 diagnoses reported in 2020 [1]. It is responsible for an estimated 500,000 new cases and 200,000 deaths annually on a global scale [2]. Epidemiological studies have consistently reported a difference in incidence between sexes, with BC occurring more frequently in men than in women [3,4,5]. While advanced age remains the most significant risk factor for BC, with the average age of diagnosis falling between 70 and 84 years [6], other risk factors contribute to its high incidence. These include smoking, workplace exposure to chemicals, and genetic predisposition [7,8,9].

For muscle-invasive bladder cancer (MIBC), which accounts for approximately 25% of newly diagnosed cases, and considering the emerging therapies [10,11,12], radical cystectomy with urinary diversion remains the gold standard treatment [13]. This procedure involves the complete removal of the bladder and the creation of a new pathway for urine to exit the body. Ureterocutaneostomy, a type of urinary diversion where ureters are directly connected to an opening in the abdominal wall, is one of the surgical options for patients undergoing radical cystectomy. In Europe, approximately 700,000 people are living with a stoma, including urostomies [14]. In Italy, survey results indicate that more than 70,000 individuals have a stoma [15,16].

### 1.1. Clinical Impact of Ureterocutaneostomy

Undergoing ureterocutaneostomy surgery can have significant implications for a patient’s quality of life [17,18]. Studies have shown that individuals with urostomies often face challenges in various aspects of daily living, including bodily comfort, social interactions, financial concerns, and intimacy [8,19,20,21,22,23]. These challenges can stem from issues such as leakage concerns, urine odor, skin irritation, recurrent infections, and dependency on pouches [14,24,25]. Indeed, individuals with stomas, including those with urostomies, often experience a range of psychological challenges that can impact their overall well-being [26,27,28,29,30].

### 1.2. Quality of Life in Stoma Patients

A systematic review [31] found that ostomy patients frequently report issues such as depression, anxiety, and social isolation. These mental health concerns correlate with a global decreased Health-related Quality of Life (HRQOL) [32,33]. Furthermore, patients with urostomies reported lower scores on measures of psychological well-being compared to those who underwent other types of urinary diversion procedures [34].

In particular, depression has emerged as a significant mental health concern among ostomy patients. A recent systematic review and meta-analysis [35] found that the pooled prevalence of depression in stoma patients was 26.7%, significantly higher than in the general population, with rates varying depending on factors such as time since surgery and the presence of complications [36]. The impact of depression on quality of life in this population is substantial [35,37], affecting adherence to self-care regimens, even from a longitudinal perspective [38,39,40].

Among the various factors influencing the mental health of urostomy patients, the experience of illness and the presence of a stoma represents a visible alteration to body appearance, which can profoundly impact an individual’s self-perception and self-esteem [41,42,43,44,45]. Body image (BI), which encompasses perceptions of physical appearance and functionality, involves affective dimensions such as feelings, thoughts, and attitudes towards the body as a representation of personal identity [46,47,48], which can be endangered by ostomy [49,50,51,52,53]. Specifically, poor BI may play a significant role in the mental health distress and quality of life of ostomy patients due to significant concerns about changes in their bodily appearance and struggles with acceptance of their altered BI, including perceived discomfort while adjusting to the pouching system [54,55,56,57]. In this context, stoma acceptance is a crucial factor in the psychological adjustment and overall well-being of patients with a stoma, MIBC patients with ureterocutaneostomy included. Acceptance refers to the process by which individuals come to terms with their stoma and integrate it into their self-concept and daily life [58,59]. Higher levels of stoma acceptance are associated with improved mental health outcomes, better quality of life [58,60], and independent self-management of the stoma [61,62]. Research has shown that enhancing acceptance may improve adjustment and prevent BI distress, improving well-being and self-efficacy [36]. This evidence aligns with findings indicating that patients with higher levels of stoma acceptance experience better mental health outcomes and enhanced social functioning [63]. Additionally, Jin et al. [64] identified that feelings of self-disgust play a significant role in adjustment to stoma and mental health; thus, addressing acceptance can further enhance mental health in ostomy patients.

### 1.3. The Current Study

**Figure 1:**
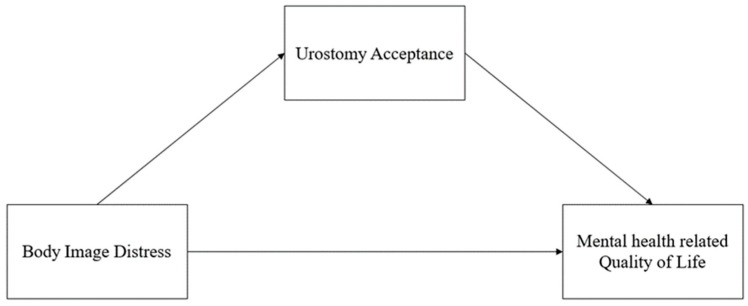
Theoretical diagram of mediation model proposed in this study.

Despite these premises, previous studies have not investigated the mediating role of stoma acceptance in patients diagnosed with MIBC who have undergone ureterocutaneostomy regarding BI distress and mental health-related QoL. Therefore, the aim of the current study was to test a series of hypotheses that integrate BI distress, stoma acceptance, and mental health in a mediation model (Figure 1). Firstly, our hypothesis suggested that BI distress negatively affects mental health. Secondly, we proposed that acceptance is positively associated with mental health. Thirdly, we hypothesized that acceptance mediates the relationship between BI distress and mental health.

## 2. Material and Methods

### 2.1. Procedures and Participants

In this study, we used a single-center cross-sectional survey to collect data from patients recruited at the Urology Department of the University Hospital of Naples Federico II. Participants were considered eligible if they (1) were over 18 years of age, (2) had a histologically confirmed MIBC diagnosis that required surgical intervention with radical cystectomy and ureterocutaneostomy, and (3) were able to read, understand, and agree to give informed consent and complete the survey autonomously. Patients were excluded if they (1) had cognitive impairment affecting survey comprehension, (2) presented acute psychiatric conditions requiring treatment, (3) were in terminal illness or palliative care conditions, (4) were unable to provide informed consent, or (5) were non-Italian speaking. Based on these criteria, 73 MIBC patients were enrolled in this study between April 2023 and February 2024. All participants underwent surgery at the aforementioned hospital and, since discharge, had checked into our clinic periodically (once/twice per month) for ureteral catheter replacements and routine examinations. During the visits, they were asked to participate in this survey. To minimize social desirability bias, the clinicians involved in data collection informed participants that all investigators were ethically obligated to anonymize data from patients’ medical records in order to safeguard their identities, especially from healthcare providers who might have recognized them.

All participants provided their consent to participate and were informed of their right to withdraw from the survey in any circumstance and for any cause. This study was designed with respect for the principles of the Declaration of Helsinki on Ethical Principles for Medical Research Involving Human Subjects, European Union regulations 2016/679 (general data protection regulation—GDPR) and 2018/1725 (data protection obligations for the EU). Furthermore, this study was approved by the ethical committee of the School of Medicine and Surgery of the University of Naples Federico II (protocol number 261/2019).

### 2.2. Measures

#### 2.2.1. Socio-Demographic and Clinical Characteristics

Socio-demographic and clinical data were collected from patients’ reports, including gender, age, education level (≤high school vs. ≥university), marital status (with partner vs. without partner), time since surgery, stoma care and management (autonomous vs. non-autonomous), and urine/blood leakage from urostomy.

#### 2.2.2. Body Image Distress

In order to assess levels of BI distress, we used the validated Italian version of the Body Image Scale (BIS) [65,66], a specific tool to assess cancer patients’ perceptions and attitudes towards their BI. This scale comprises 10 items rated on a 4-point Likert scale, ranging from 0 (not at all) to 3 (very much), which covers dimensions such as affective (e.g., feeling attractive), behavioral, (e.g., experiencing difficulty viewing themselves naked, avoiding social occasions), and cognitive (e.g., satisfaction with appearance). The Cronbach’s α for the current sample was 0.91.

#### 2.2.3. Psychological Adjustment to Urostomy

Focusing on the psychological adjustment to urostomy, we applied the validated Italian version of the Ostomy Adjustment Inventory (OAI-23) [67,68]. This scale consists of 23 items rated on a 5-point Likert scale, ranging from 0 (strongly disagree) to 4 (strongly agree). The OAI-23 measures three dimensions of adjustment that are acceptance of the ostomy, negative emotions, and social engagement. In this regard, we focused on the acceptance subscale to assess the extent to which patients had come to terms with their urostomy. The Cronbach’s α of the acceptance subscale for the current sample was 0.73.

#### 2.2.4. Mental Health-Related Quality of Life

The Short Form (SF) 12 Health Survey [69,70] was administered to evaluate perceived health-related QoL. The SF-12 consists of 12 items that cover eight domains regarding physical, emotional, and social impairment. Scores from the SF-12 are computed to generate two principal summary measures regarding the Physical Component (PCS12) and the Mental Component (MCS12). Specifically, to assess mental health-related QoL, we referred to the MCS12 index, which combines scores from the mental health domains: vitality, social functioning, emotional role, and mental health. The Cronbach’s α for the current sample was 0.84.

### 2.3. Statistical Analysis

Statistical analyses were performed using the Statistical Package for the Social Sciences (SPSS 29). First, participants’ clinical characteristics, descriptive statistics, and bivariate correlations between BI distress, urostomy acceptance, and mental health-related QoL were calculated. Then, a mediation model analysis was conducted to test the direct and mediating effects of BI distress and urostomy acceptance on mental health-related QoL. Before performing the analyses, the continuous variables were centered. In this model, gender, age, time since stoma surgery, leakage, and autonomy were included as control variables. The statistical package for the social sciences (SPSS) with PROCESS Macro (Model 4) [71] was used to assess the statistical significance of the direct and mediating effects with bias-corrected bootstrapping (10,000 samples) and 95% confidence intervals (CIs). According to Hayes [71], the indirect effect can be considered significant if the upper and lower boundaries of the bias-corrected 95% CI do not comprehend value zero.

## 3. Results

### 3.1. Participant Characteristics

In total, 73 MIBC patients participated in the survey. Among the participants, 53 (72.6%) were male and 20 (27.4%) were female. Ages ranged from 42 to 81 years (mean = 69.56, SD = 8.92), with most having an education level of high school or lower (n = 64; 87.7%) and being in a stable relationship (67.1%). Concerning their clinical status, the mean time since surgery was 5.58 years (SD = 3.63, range = 1–15 years). Additionally, 54 participants (74%) experienced leaking from the stoma at least one time per month and 46 (63%) stated that they were mostly non-autonomous in the hygienic care and management of their stoma (Table 1).

### 3.2. Descriptive and Bivariate Correlations

Through a descriptive analysis of the collected sample, 82.2% of participants reported BI distress scores exceeding the cut-off value of 11 [65,66], indicating a prevalent concern and struggle with their BI. Furthermore, 71.2% of the sample scored ≤42 on the MCS12 index; scores less than or equal to this threshold are indicative of depression [68], suggesting a substantial impairment of mental health within the sample studied. Also, Pearson correlation results showed a significant interrelation between all the variables. Specifically, BI distress correlated negatively with QoL and acceptance. Instead, acceptance correlated strongly and positively with mental health. Means, standard deviations, ranges, and bivariate correlations between the variables analyzed are shown in Table 2.

### 3.3. Direct and Indirect Effects of Body Image Distress and Urostomy Acceptance on Mental Health-Related Quality of Life

**Figure 2:**
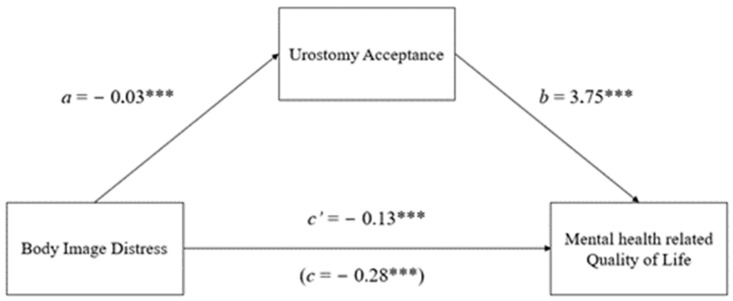
Mediated effect of urostomy acceptance on the relationship between body image distress and mental health-related quality of life. *** *p* < 0.001. All values are beta coefficients.

First and foremost (Figure 2), the analysis yielded results that supported our first hypothesis since BI distress was negatively associated with urostomy acceptance (*β* = −0.03, standard error (SE) = 0.01, 95% CI (−0.05, −0.01), *p* < 0.001) and negatively associated with mental health-related QoL (*β* = −0.28, standard error (SE) = 0.11, 95% CI (−0.49, −0.06), *p* < 0.001). Regarding our second hypothesis, the analysis showed that urostomy acceptance was positively associated with mental health-related QoL (*β* = 3.75, SE = 1.23, 95% CI (1.30, 6.19), *p* < 0.001). When we included urostomy acceptance as a mediator, there was a significant overall effect (*β* = −0.41, SE = 0.11, 95% CI (−0.62, −0.20), *p* < 0.001), while the direct effect remained significant, indicating a case of partial mediation and confirming our third hypothesis. Indeed, the indirect effects showed that urostomy acceptance significantly mediated the association between BI distress and mental health-related QoL (*β* = −0.13, SE = 0.06, 95% CI (−0.25, −0.03)). In addition, BI distress and urostomy acceptance explained a significant proportion of the variance in mental health-related QoL (R^2^ = 0.45). Among the covariates, gender, age, and stoma leakage were not significant predictors in either model, while time since surgery and autonomy were significant (*p* < 0.01).

## 4. Discussion

In this paper, we explored the relationship between stoma acceptance, BI distress, and mental health-related QoL in a sample of patients undergoing cystectomy and ureterocutaneostomy due to MIBC. Regarding the first hypothesis, the current study corroborates a significant association between BI distress and poorer mental health outcomes, which aligns with White’s model (2000) about BI alterations linked to poorer psychosocial outcomes, particularly for individuals experiencing evident bodily changes [45,69,70]. Thus, BI distress exacerbates emotional distress and triggers negative cognitions towards one’s own appearance, particularly for patients with a permanent stoma [23,53]. Our results fit with findings that recognize BI distress as a critical risk factor among patients undergoing stoma surgery [35,49,72]. Indeed, stoma formation significantly affects an individual’s bodily sensations and functionality, disrupting the perceived unity between the altered body and self-concept. Furthermore, alteration in BI due to neoplastic disease elicits a complex emotional response characterized by uncertainty [73], perceived stigma [64], and deviation from beauty standards [74]. Consequently, patients often experience diminished self-esteem and self-efficacy accompanied by feelings of frustration and helplessness [28,35,36]. These findings are consistent with our results about the association between BI distress and stoma acceptance, where feelings of self-disgust towards stoma and physical appearance can lead to worsened stoma acceptance [64].

According to our second hypothesis, we found that stoma acceptance is positively associated with mental health-related QoL. This result is consistent with well-established findings highlighting that stoma acceptance is a crucial process to enhance well-being [58,75,76,77,78]. This evidence has also been widely confirmed in cancer research underscoring adjustment strategies for diagnosis, surgery, and non-operative therapies [79,80,81]. A valuable framework to understand this association is the Triple A Model [78], which emphasizes the role of acceptance as the initial stage of a dynamic process. Through acceptance, patients can transition from a state of perceived loss to active engagement, achieving adjustment to the new body feature and autonomy in self-care [42,58,82]. This process involves an open stance towards adverse experiences without trying to eliminate them, promoting greater psychological flexibility [83]. A recent study [84] demonstrated that greater stoma acceptance is correlated with lower levels of social isolation and psychological distress, coherent with evidence showing a positive association between acceptance and emotional resources [64,85,86]. Thus, our results confirm that acceptance can act as a protective mechanism against the negative effects of stoma-related difficulties.

Lastly, the results confirmed the third hypothesis since stoma acceptance partially mediated the effect of BI distress on mental health-related QOL. To our knowledge, this is the first study to test this mediation model in a specific population of MIBC patients undergoing radical cystectomy and ureterocutaneostomy. This model can be understood in light of recent research that considered acceptance as a mediator between stressors and health outcomes, both for colorectal cancer patients and other medical conditions [84,87,88,89]. This evidence corroborates the finding that urostomized patients with high BI distress levels may benefit from stoma acceptance to enhance their quality of life and reduce the likelihood of developing psychological distress. This result may be explained considering the risk of social isolation, as observed by [84], who reported that ostomized patients face feelings of shame, social pressure, and disability stigma, all of which negatively affect their quality of life. The relationship between BI, mental health, social isolation, and stigma in urostomized patients appears to be intricate and multifaceted. The stigma associated with having a stoma can lead to BI dissatisfaction and social isolation as patients may withdraw from social interactions due to perceived discomfort. This isolation, in turn, can exacerbate mental health issues and, in turn, poor mental health may further reinforce social withdrawal and heighten sensitivity and shame regarding the altered body image, creating a detrimental cycle. By accepting their stoma, patients may be more confident and inclined to engage in social interactions, reducing isolation and its associated mental health risks [84]. These findings align with and expand upon our understanding of the role of acceptance in promoting psychological well-being among patients with stomas, considering that stoma acceptance is an important mediator in order to foster agency and self-efficacy [64,89]. Instead, patients who do not accept their stoma may exacerbate negative perceptions of themselves and their bodies, resulting in self-conscious feelings, avoidant behaviors, and perceived public discrimination [64,84]. Furthermore, our findings align with recent research on the mediating role of acceptance in many health contexts. Jin et al. [64] demonstrated that pain acceptance partially mediated the effects of perceived injustice on pain intensity, physical disability, and depressive symptoms. In line with these premises, this study corroborates the finding that acceptance plays a crucial role across different medical conditions, specifically those involving permanent alterations of body function and/or appearance, which may endanger psychosocial well-being and overall quality of life. Supporting this notion, Qi et al. [88] found that illness acceptance mediated the relationship between the health locus of control and symptom distress in acute leukemia patients. The consistency of our findings, corroborated by these studies, underscores the importance of the psychological processing of acceptance in medical conditions, particularly for stoma patients. Our results imply that fostering stoma acceptance may be a crucial intervention target to mitigate the negative impact of BI distress on mental health and overall quality of life, addressing both cognitive (e.g., health beliefs, expectations) and emotional (e.g., rejection, adjustment) factors [88,90]. Furthermore, covariates in the mediation model provided important insights, indicating that those who underwent surgery several years ago and perceived themselves as autonomous, confident, and proficient in stoma care and demonstrated better mental health outcomes [59,76,91,92,93]. In conclusion, it is imperative to recognize that the prevention, both secondary and tertiary, of negative mental health outcomes through stoma acceptance is a path that starts from a complex interplay between individual, psycho-social, and contextual dimensions, resulting in a better understanding of patients’ support needs [94]. Discussing stoma-related possibilities and issues at an early stage of the disease may prevent the development of post-traumatic stress disorder following surgery and foster patient autonomy [95,96]. This proactive approach is crucial as recent research has highlighted the potential risks associated with delegating stoma care to caregivers in terms of mental health outcomes. Indeed, depressive symptoms have been positively correlated with caregiver contribution to self-care maintenance [97], while the progressive acceptance of the stoma, promoted by stoma therapists, contributes to patients’ progression in developing self-care strategies and substantially better adaptation [78,98].

Based on our findings, we propose several practical recommendations for healthcare providers to support stoma acceptance. First, early psychological screening should be implemented to identify patients at risk for poor adjustment, with particular attention to body image concerns [36]. Second, stoma care nurses should integrate psychological support with technical training, acknowledging that acceptance develops alongside practical management skills [78]. Third, healthcare providers should consider implementing structured acceptance-based interventions that (a) provide peer support opportunities with confident long-term stoma patients, (b) involve family members in the acceptance process [94], and (c) offer progressive exposure to stoma management [98].

Additionally, the timing of interventions appears crucial as our results showed that time since surgery was a significant predictor. Therefore, we recommend intensifying psychological support in the early post-operative period while maintaining long-term follow-up to monitor adjustment [76]. The significant association between autonomy and better mental health outcomes suggests that healthcare providers should prioritize patient empowerment in stoma care, gradually building confidence and independence [92,93].

## 5. Conclusions

This study demonstrated that stoma acceptance partially mediates the relationship between body image distress and mental health-related quality of life in MIBC patients with ureterocutaneostomy. Body image distress negatively impacted mental health outcomes, while stoma acceptance emerged as a protective factor. These findings suggest that healthcare professionals should implement interventions targeting stoma acceptance to prevent body image disruption and promote psychological adjustment. The integration of acceptance-based approaches within standard stoma care could improve mental health outcomes in this patient population. By fostering stoma acceptance, healthcare providers can enable patients to develop adaptive coping strategies, resulting in improved mental health outcomes despite body image challenges. These findings underscore the importance of holistic stoma care, integrating psychological interventions with physical management, including acceptance-based therapies and cognitive restructuring techniques, to better support patients in adapting to their new body reality. Future research should focus on developing and testing structured acceptance-based interventions, particularly in the early post-operative period, and evaluating their long-term impact on patients’ psychological adaptation and self-management skills.

### Limitations

This study has several limitations that must be acknowledged while interpreting the results. Firstly, its cross-sectional design precludes firm conclusions about the temporality and causality of the relationships among variables. Future research should employ multi-center longitudinal designs to establish cause-and-effect relationships and overcome current limitations.

Secondly, the sample was relatively small and recruited from a single hospital, which limits the generalizability of the findings; thus, the results may not fully represent the broader population of MIBC and urostomized patients, particularly those from different age groups, socioeconomic backgrounds, or healthcare settings. Moreover, in the current study, the average age of the sample limits the applicability of the findings to other age groups, such as younger individuals, who may experience different challenges that can enhance or compromise stoma acceptance. Age-related differences in BI concerns and psychological mechanisms could lead to different mental health outcomes, which future studies should explore. Additionally, as expected in a population with cancer history, participants in this study reported relevant BI distress and mental health issues, which may have influenced their perceptions of their stoma and related challenges. It is plausible that psychological distress led to an overestimation of the impact of the stoma, thereby skewing the results. Indeed, another limitation is the potential influence of unmeasured variables, such as comorbidities, prior mental health history, cancer-related emotional distress, and social support, which were not controlled in this study but could significantly affect the relationships among the variables examined. Moreover, some clinical variables like leakage were measured only dichotomously (presence/absence), potentially failing to capture important nuances in severity and frequency that could better predict psychological outcomes. These factors should be considered in future research to provide a more comprehensive understanding of the determinants of mental health in urostomized patients in order to develop tailored and effective interventions.

## Figures and Tables

**Table 1:** Socio-demographic characteristics of the sample.

	N (%)	Mean	SD	Min/Max	Mean	IQR
Age	73	69.56	8.92	42–81	-	-
Gender	73	-	-	-	-	-
Male	53 (72.6%)	-	-	-	-	-
Female	20 (27.4%)	-	-	-	-	-
Education level	73	-	-	-	-	-
≤High school	64 (87.7%)	-	-	-	-	-
≥University	9 (12.3%)	-	-	-	-	-
Partner	73	-	-	-	-	-
Yes	49 (67.1%)	-	-	-	-	-
No	24 (32.9%)	-	-	-	-	-
Stoma care	73	-	-	-	-	-
Autonomous	27 (37%)	-	-	-	-	-
Non-autonomous	46 (63%)	-	-	-	-	-
Time since surgery (years)	73	5.58	3.63	1–15	5	6
Leaking from the stoma	73	-	-	-	-	-
Yes	54 (74%)	-	-	-	-	-
No	19 (26%)	-	-	-	-	-

**Table 2:** Descriptive statistics and bivariate correlations between body image distress, mental health-related quality of life, and acceptance of urostomy. Note: M = mean; SD = standard deviation; *** = *p* < 0.001.

	1	2	3	Ranges	M ± SD
1. Body Image Distress	-			1–30	19.5 ± 7.1
2. Mental Health-Related QoL	–0.492 ***	-		23.43–53.96	37.76 ± 6.9
3. Acceptance of Urostomy	–0.445 ***	0.491 ***	-	0.64–3.18	2.11 ± 0.58

## Data Availability

The datasets generated and analyzed during the current study are available from the corresponding author [C.C.R.] upon reasonable request.
